# Palmitate lipotoxicity is closely associated with the fatty acid-albumin complexes in BV-2 microglia

**DOI:** 10.1371/journal.pone.0281189

**Published:** 2023-04-20

**Authors:** Yanzhuo Yang, Qingting Yu, Bin Li, Zuisu Yang, Shuai Zhang, Falei Yuan

**Affiliations:** 1 School of Food and Pharmacy, Zhejiang Ocean University, Zhoushan, China; 2 Zhoushan Institute for Food and Drug Control, Zhoushan, China; University of North Dakota School of Medicine and Health Sciences, UNITED STATES

## Abstract

Palmitic acid (PA) is considered a major contributor to the inflammation in many metabolic diseases; however, this role has been questioned recently for the complicated procedures in preparing PA-bovine serum albumin (BSA) complex. This study is aimed to evaluate the effect of PA-BSA complexing methods on cell viability and inflammatory responses of BV-2 cells. Three commercially available BSA brands and two types of solvents were compared for their effects on the expression of inflammatory cytokines. Three commonly used proportions of PA-BSA were tested for cell viability and inflammatory responses. We found that all the three types of BSA were proinflammatory. Both ethanol and isopropanol dampened inflammation except that 1% isopropanol treatment increased the IL-1β level by 26%. When reducing the BSA content in PA-BSA solutions from 3:1 to 5:1, a marked increase in cell viability (11%) was seen. To our surprise, reducing BSA content in PA-BSA solutions from 5:1 to 10:1 decreased cell viability by 11%. The 5:1 group exhibited the lowest inflammatory profile. Either PA-BSA or BSA alone increased the entry of LPS to the cytosol, which further caused pyroptosis. In summary, we found 5:1 (PA:BSA) to be the best binding ratio for studying inflammation in BV-2 microglia. The presence of LPS in the cytosol in the context of BSA might be the reason for confounding results from palmitate studies.

## Introduction

PA is one of the most abundant fatty acids in the human body. Long-chain saturated fatty acids like PA can directly act on microglia, causing inflammation and dysregulation in the hypothalamus [1]. PA levels are elevated in the cortex of AD patients [2], and early-stage hypothalamic dysfunction can appear even prior to cognitive deficits [3]. The functions of PA in the central nervous system are difficult to study because there is an abundance of PA in diet, as well as PA produced from de novo lipogenesis. For neural cell models, PA is usually incubated with bovine serum albumin (BSA) for two reasons: (1) to increase the aqueous solubility of PA, and; (2) to mimic physiological conditions regarding long-chain fatty acid transportation. BSA is composed of six high-energy binding sites in addition to many weak binding sites [4]. Under normal physiological conditions, the ratio of fatty acids bound to BSA is between 1:1 and 2:1 [5, 6]. In pathological states, the ratio can reach as high as 6:1 [7, 8]. For this reason, most in vitro studies have used a free fatty acid (FFA) to BSA ratio that ranges from 3:1 to 6:1. Some studies have used a ratio as high as 10:1, without detailing the reason for the excessive fatty acid [9–12]. The FFA to BSA ratio is frequently not mentioned despite the key role of BSA in FFA preparations [13–17]. Recent studies have shown that BSA is able to induce inflammation in macrophages [18]. BSA, being a protein, may also have other biological effects on lipotoxicity models [19].

Two forms of PA, palmitic acid and sodium palmitate, are often used in cellular lipotoxicity studies. For the FFA form, which has a melting point of 62.9°C, ethanol (or alternatively isopropanol) is frequently used as a solvent to increase the solubility when conjugating PA with BSA in aqueous solutions [9, 19–21]. Ethanol is reported as a quenching substance for proinflammatory cytokines induced in macrophages, especially tumor necrosis factor-α (TNF-α) and interleukin-6 (IL-6) expressions [18]. However, it has also been found to promote inflammatory responses in BV-2 microglia [22].

Microglia are recognized as being innate resident immune cells in the central nervous system, and play an important role similar to that of macrophages [23]. Microglia neuroinflammation has been reported in the pathogenesis and progression of various kinds of neurodegenerative diseases [24, 25]. Microglia can be activated quickly after stimulation and have been shown to secrete cytokines such as tumor necrosis factor TNF-α, IL-6, interleukin-1β (IL-1β), and inducible nitric oxide synthase (iNOS) that promote the occurrence and development of the inflammatory response [26].

Confounding results are often obtained in PA-related lipotoxicity models. Although PA has been generally reported as toxic to microglia, Tracy et al. (2013) did not observe any changes in the TNF-α, IL-6, and IL-1β levels after treating BV-2 microglia with 125 μM PA dissolved in ethanol [16]. Additionally, PA is generally considered harmful to neuronal cells at 100 μM, however, reports in the literature show concentrations of 200 μM as being protective, and a relatively low concentration of 25 μM giving rise to a significant amount of apoptosis [11, 14]. These results may highlight the importance of the protocol in preparing FFA/BSA complex, as this may be the reason for the divergence in results [27]. Whether PA is indeed proinflammatory is still under debate due to the complexity of experimental protocols [18, 19]. With this study, we aim to evaluate the effects of PA on inflammation and cell viability in BV-2 microglia.

## Methods and materials

### Cell culture

The murine BV-2 cells (identification by STR) used in all experiments were purchased from Procell Inc., Wuhan, China. The cells were maintained in high-glucose Dulbecco Modified Eagle’s Medium (DMEM, HyClone, USA) and supplemented with 10% heat-inactivated fetal bovine serum (Sijiqing, Hangzhou, China) and 1% penicillin-streptomycin solution (Procell, Wuhan, China) at 37°C and 5% CO_2_ in a humidified incubator. For all experiments, BV-2 cells were used at 70 to 85% confluency. Before treatment, the medium was replaced with DMEM containing sodium palmitate (Aladdin, Shanghai, China). Low-endotoxin and fatty acid-free bovine serum album (BSA) was purchased from three different manufacturers (Sigma-Aldrich, St. Louis, MO, USA; Cat#SRE0098, endotoxin < 3EU/mg) (Beyotime, Shanghai, China. Cat#ST025, endotoxin < 0.01EU/mg) (Lanso, Zhejiang, China; Cat#AS33654, endotoxin < 0.1EU/mg). Lipopolysaccharide was obtained from Beyotime Inc., Shanghai, China.

### Preparation of fatty acid-BSA complex

A 50mM stock solution of sodium palmitate (PA) was prepared fresh with distilled water at 70°C. The stock solution was then diluted 25-fold into preheated (37°C) DMEM containing the corresponding proportion of BSA (3:1 to 10:1). The 2mM PA/BSA solution was then heated at 37°C with shaking for 1h to allow for complex formation. The 2mM PA-BSA complex solution was diluted with DMEM to 100 μM and filtered through 0.22 μm pore membrane filters.

### Cell viability assay

BV-2 cells were seeded onto plates (8×10^4^ cells/well for 24-well plate) and cultured for 24 h, after which the medium was replaced by serum-free medium containing either PA-BSA complexed solution or vehicle (containing only the equal proportion of BSA solution). After 24 h, cells were incubated for 2 h with the cell counting kit-8 (CCK-8, ApexBio, Houston, TX, USA), added by ten percent of the volume of the medium. Following this, the absorbance at 450 nm was measured with a microplate reader (SpectraMax M2, Molecular Devices, CA, USA). Cell viability is expressed as a percentage of the control.

### Quantitative PCR (qPCR) analysis

After BV-2 cells were treated with BSA, PA-BSA or lipopolysaccharide (LPS, 1 μg/mL) for 24 h, they were washed with sterile phosphate-buffered saline before all the RNA was extracted and purified using the RNAeasy^™^ Animal RNA Isolation Kit (Beyotime, Shanghai, China) following the manufacturer’s instructions. Complementary DNA was generated with HiScript III RT SuperMix for qPCR (Vazyme, Nanjing, China). Real-time qPCR was performed using 2X SYBR Green qPCR Master Mix (ApexBio, Houston, TX, USA) in the LineGene detection system (FQD-48A, Hangzhou Bioer Inc., Zhejiang, China) with the following PCR conditions: 95°C for 2 minutes, 40 cycles for 15 sec at 95°C, 55°C for 30 sec, and 72°C for 30 sec. The primers used for PCR are shown in Table 1. GAPDH was used for normalization and relative quantitation was carried out by deducting the ΔCq for the experimental group from the average ΔCq of the control group [28].

**Table 1:** Primers and their sequences used in this study.

Name of primers	Primer sequences (5’- 3’)
GAPDH Forward	TCACCACCATGGAGAAGGC
GAPDH Reverse	GCTAAGCAGTTGGTGGTGCA
TNF-α Forward	TAGCCAGGAGGGAGAACAGA
TNF-α Reverse	CCAGTGAGTGAAAGGGACAGA
IL-6 Forward	ACCAAGACCATCCAATTCATC
IL-6 Reverse	CTGACCACAGTGAGGAATGTC
IL-1β Forward	TACATCAGCACCTCACAAGC
IL-1β Reverse	AGAAACAGTCCAGCCCATACT
TLR4 Forward	CAAGAACCTGGACCTGAGCTTTA
TLR4 Reverse	GATTTGTCTCCACAGCCACCAG
iNOS Forward	GGAATCTTGGAGCGAGTTGTGGAT
iNOS Reverse	CCTCCAATCTCTGCCTATCCGTCT

### Immunofluorescence staining

BV-2 cells were treated with BSA or PA-BSA for 4 h and then washed with PBS twice. After fixation with 4% paraformaldehyde (PFA) for 15 min, cells were permeated with 0.25% Triton X-100. Primary antibodies for detecting the N terminal of gasdermin D (GSDMDC1, Novus, NBP2-33422, 1:200) and lipid A (Abcam, ab8467, 1:100) were incubated with cells overnight at 4°C. After washing with PBS three times, cells were incubated with Alexa Fluor 594-AffiniPure Goat Anti-Rabbit IgG and Fluorescein (FITC)-AffiniPure Goat Anti-Mouse IgG (Jackson ImmunoResearch, 111-585-003 and 115-095-003) at room temperature for 1 h. Finally, nuclei were counterstained with 4’,6-diamidino-2-phenylindole (DAPI). Images were then captured under a fluorescence microscope (OLYMPUS BX41, Japan).

### Western blot

BV-2 cells were ultrasonically fragmented and lysed with radioimmunoprecipitation assay buffer (RIPA, Beyotime, Shanghai, China) on ice for 30 min. The extracted protein concentration was determined using the BCA protein assay kit (Solarbio, Beijing, China). Proteins were separated via SDS-PAGE and then transferred to a polyvinylidene difluoride (PVDF) membrane. After being blocked with 5% BSA for 2 h, the membranes were incubated with the primary antibodies (TNF-α, Cat#17590, Proteintech; IL-6, Cat#66146, Proteintech; IL-1β, Cat#12507, Cell Signaling Technology; iNOS, Cat#AF7281, Beyotime; TLR4, Cat#66350, Proteintech; α-Tubulin, Cat#T9026, MilliporeSigma) at ≥ 1/1000 dilutions at 4°C overnight, and then incubated with secondary antibodies conjugated with horseradish peroxidase (Cat#111-035-003, Cat#115-035-003, Jackson ImmunoResearch) at 1/5000 dilution for 1 h at room temperature. Finally, the protein bands were detected using BeyoECL Star (Beyotime, Shanghai, China) and quantified using the Alphaview SA software for Fluor Chem FC3 (ProteinSimple, San Jose, CA, USA).

### Endotoxin detection

The endotoxin of the cell culture medium was detected using the microplate quantitative chromogenic matrix method and following the instruction of Limulus Kit for Endotoxin Detection (Cat#EC64405S, Bioendo Technology, Xiamen, China).

### Statistical analyses

Each experiment in this study was carried out with three or more replicates. All quantified data are shown as mean ± SD and analyzed by GraphPad Prism 9 software (GraphPad Software, CA, USA). Data were analyzed with one-way analyses of variance (ANOVA) and followed by Tukey’s or Dunnett’s multiple comparisons test to determine the differences between groups.

## Results

### BSA has proinflammatory effects and the BSA-induced microglial immune response is dose-dependent

**Fig 1:**
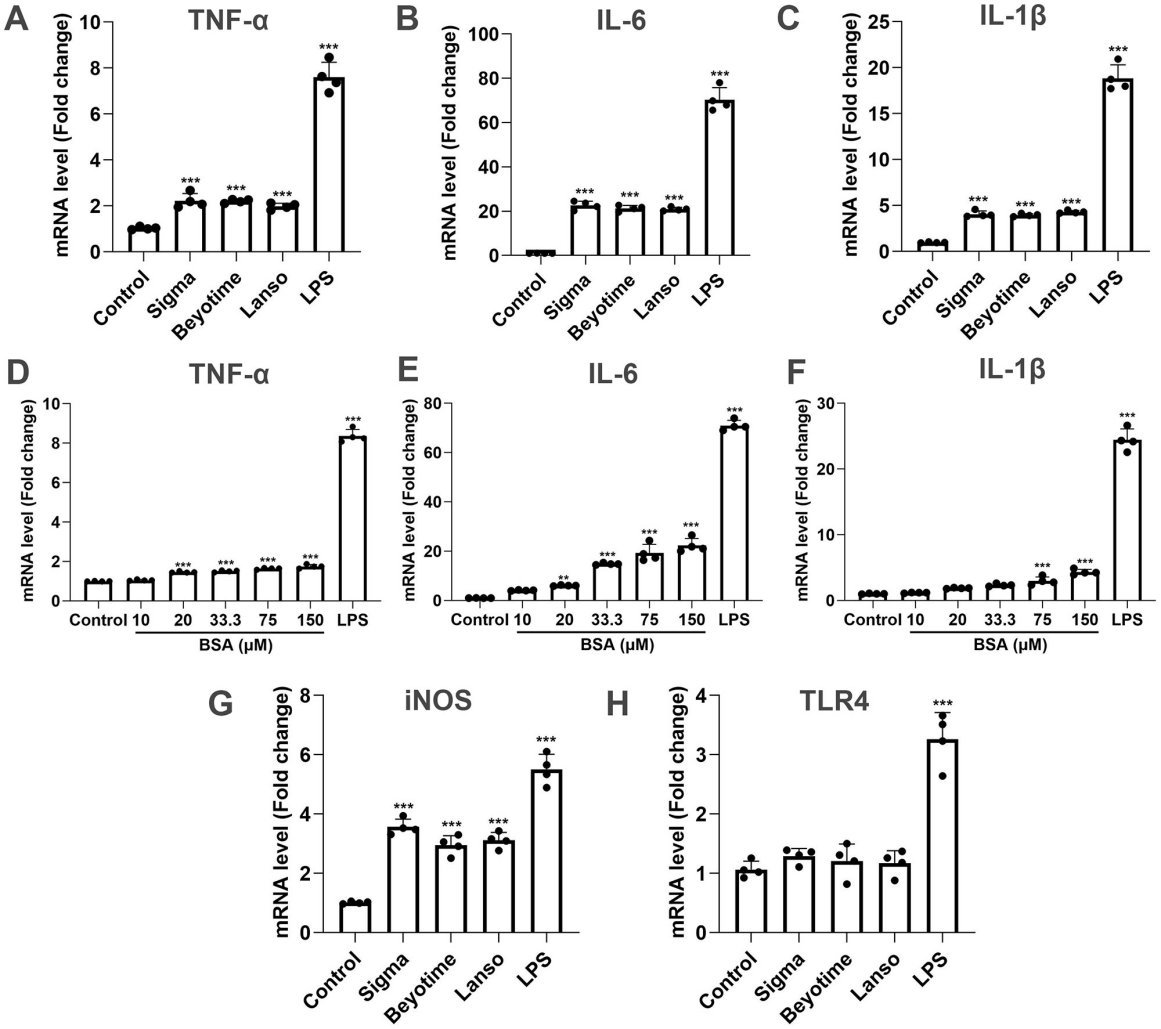
Effect of three types of BSA brands and different concentrations of BSA on inflammatory responses. (A-C, G, and H) BSA brands (150 μM) from Sigma, Lanso, and Beyotime were compared by adding to BV-2 cells for 24 h, and all three promoted TNF-α, IL-6, IL-1β, and iNOS mRNA expressions, but much lower than these induced by LPS (1 μg/mL). The change of TLR4 expression was not significant. (D-F) 0, 10, 20, 33.3, 75, and 150 μM BSA were compared by adding to BV-2 cells for 24 h. The proinflammatory effect of Lanso BSA is dose-dependent. **p < 0.01, ***p < 0.001 by one-way ANOVA with Tukey’s multiple comparisons test between the three brands, and Dunnett’s multiple comparisons test compared with the control group.

To investigate whether BSA has a proinflammatory effect on microglia, we compared three commercially available BSA brands: Sigma, Beyotime, and Lanso. All three induced the expression of inflammatory cytokines TNF-α, IL-6, IL-1β, and iNOS except the TLR4 level. (Fig 1A–1C, 1G, and 1H). There were no significant differences in the mRNA expression among the different BV-2 cell inflammatory cytokines. Lanso was selected for use in the following experiments as it induced lower inflammatory cytokine levels than the other two brands.

BV-2 cells were treated with 0, 10, 20, 33.3, 75, and 150 μM of Lanso BSA for 24 h, respectively (Fig 1D–1F). The results showed that BSA itself induced inflammation and that all three of the inflammatory cytokines we tested for exhibited a BSA dose-dependent profile. TNF-α expression rose with the increase of BSA concentrations between 20 μM and 150 μM (*p* < 0.001); however, no significant increase was seen at 10 μM (Fig 1D). The expression level of IL-6 increased significantly from 20 μM BSA, and at 150 μM (*p* < 0.001), was 22.4 times higher when compared to 0 μM (Fig 1E). Similar to the results seen with IL-6, the expression level of IL-1β increased significantly from 75 μM, with an expression level 4.3 times higher than that of 0 μM at 150 μM (*p* < 0.001, Fig 1F).

### Both ethanol and isopropanol dampen inflammation in BV-2 cells

**Fig 2:**
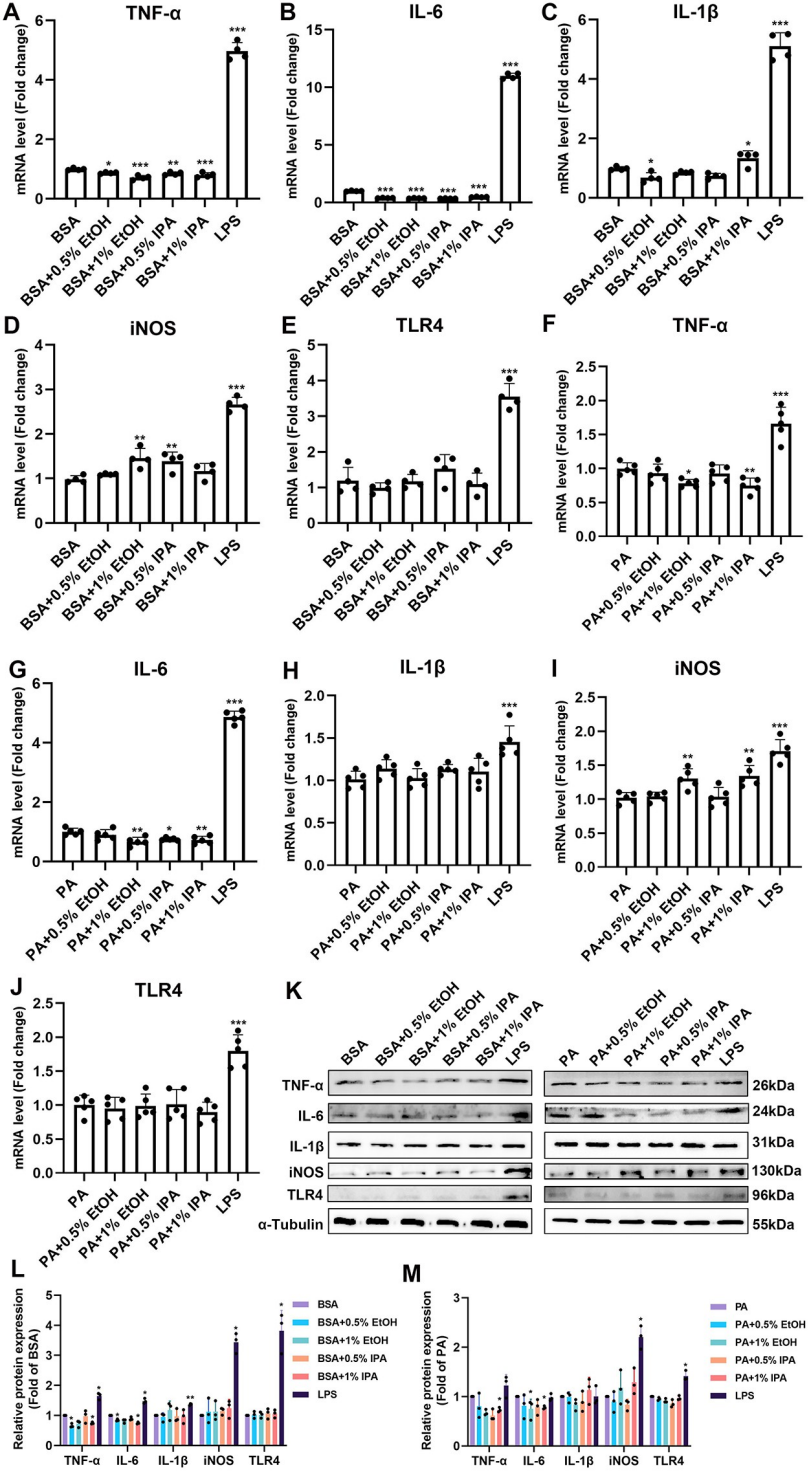
Ethanol and isopropanol both affect inflammatory responses in BV-2 cells. 0%, 0.5%, and 1% ethanol (EtOH) or isopropanol (IPA) with 33.3 μM BSA or 100 μM PA-BSA (PA) were added to each group of the cells for 24 h, respectively. (A-M) The mRNA or protein expression levels of TNF-α, IL-6, IL-1β, iNOS, and TLR4 after treatment. *p < 0.05, **p < 0.01, ***p < 0.001 by one-way ANOVA with Dunnett’s multiple comparisons test compared with the BSA or PA group.

To verify the effect of ethanol or isopropanol on microglial inflammation, we treated BV-2 cells with 33.3 μM BSA or 100 μM PA-BSA and various concentrations of ethanol or isopropanol for 24 h. As shown in Fig 2A and 2F, the mRNA fold change of TNF-α decreased by 13% (vs. BSA, *p* < 0.05), 7% (vs. PA, *p* = 0.69) after 0.5% ethanol treatment, and 29% (vs. BSA, *p* < 0.001), 22% (vs. PA, *p* < 0.05) after being treated with 1% ethanol. Compared to the group treated with plain BSA or PA-BSA, the mRNA fold change of IL-6 decreased by 61% (vs. BSA, *p* < 0.001), 10% (vs. PA, *p* = 0.51) after 0.5% ethanol treatment and 63% (vs. BSA, *p* < 0.001), 33% (vs. PA, *p* < 0.01) after being treated with 1% ethanol (Fig 2B and 2G). The same could be seen with IL-1β expression, with 0.5% ethanol having an inhibitory effect of about 31% (vs. BSA, *p < 0*.*05*). However, compared with the PA only group, ethanol had no significant effect on the IL-1β expression. A similar anti-inflammatory effect was observed in the groups treated with isopropanol. As shown in Fig 2A and 2F, the mRNA fold change of TNF-α decreased by 15% (vs. BSA, *p* < 0.01), 7% (vs. PA, *p* = 0.67) after 0.5% isopropanol treatment, and 29% (vs. BSA, *p* < 0.001), 25% (vs. PA, *p* < 0.01) after being treated with 1% isopropanol. Compared to the group treated with only BSA or PA-BSA, the expression of IL-6 was inhibited by 66% (vs. BSA, *p* < 0.001), 28% (vs. PA, *p* < 0.05) after 0.5% isopropanol treatment, and 52% (vs. BSA, *p* < 0.001), 24% (vs. PA, *p* < 0.01) after being treated with 1% isopropanol (Fig 2B and 2G). Interestingly, after being treated with 0.5% and 1% isopropanol, the mRNA fold change of the IL-1β decreased by 26% (vs. BSA, *p* = 0.70) and increased by 26% (vs. BSA, *p* < 0.05), respectively (Fig 2C). However, compared with the PA only group, isopropanol had no significant effect on the IL-1β expression (Fig 2H). Both solvents induced a significant increase of iNOS levels and yet TLR4 levels were not significant (Fig 2D, 2E, 2I and 2J).

We further analyzed the protein expression levels of the above inflammatory cytokines in the presence of BSA or PA-BSA (Fig 2K–2M). Similar to the mRNA expressions, the protein expression level of TNF-α decreased by 15% (vs. BSA, *p* < 0.05), 19% (vs. PA, *p* = 0.64) after 0.5% ethanol treatment, and decreased by 25% (vs. BSA, *p* < 0.05), 22% (vs. PA, *p* < 0.05) after 1% isopropanol treatment. As for the protein expression level of IL-6, 0.5% ethanol damped it by 34% (vs. BSA, *p* < 0.05), 1% ethanol by 33% (vs. PA, *p* < 0.05), and 1% isopropanol by 26% (vs. BSA, *p* < 0.05), 27% (vs. PA, *p* < 0.05). However, both solvents had no significant effect on the protein expressions of IL-1β, iNOS, or TLR4. Thus, it can be said that the use of ethanol or isopropanol as solvents in lipotoxicity assays may have an effect on both mRNA and protein expressions of inflammatory cytokines.

### BSA alone does not affect BV-2 cell viability, but reducing BSA content in a solution of PA-BSA complex increases cell viability

**Fig 3:**
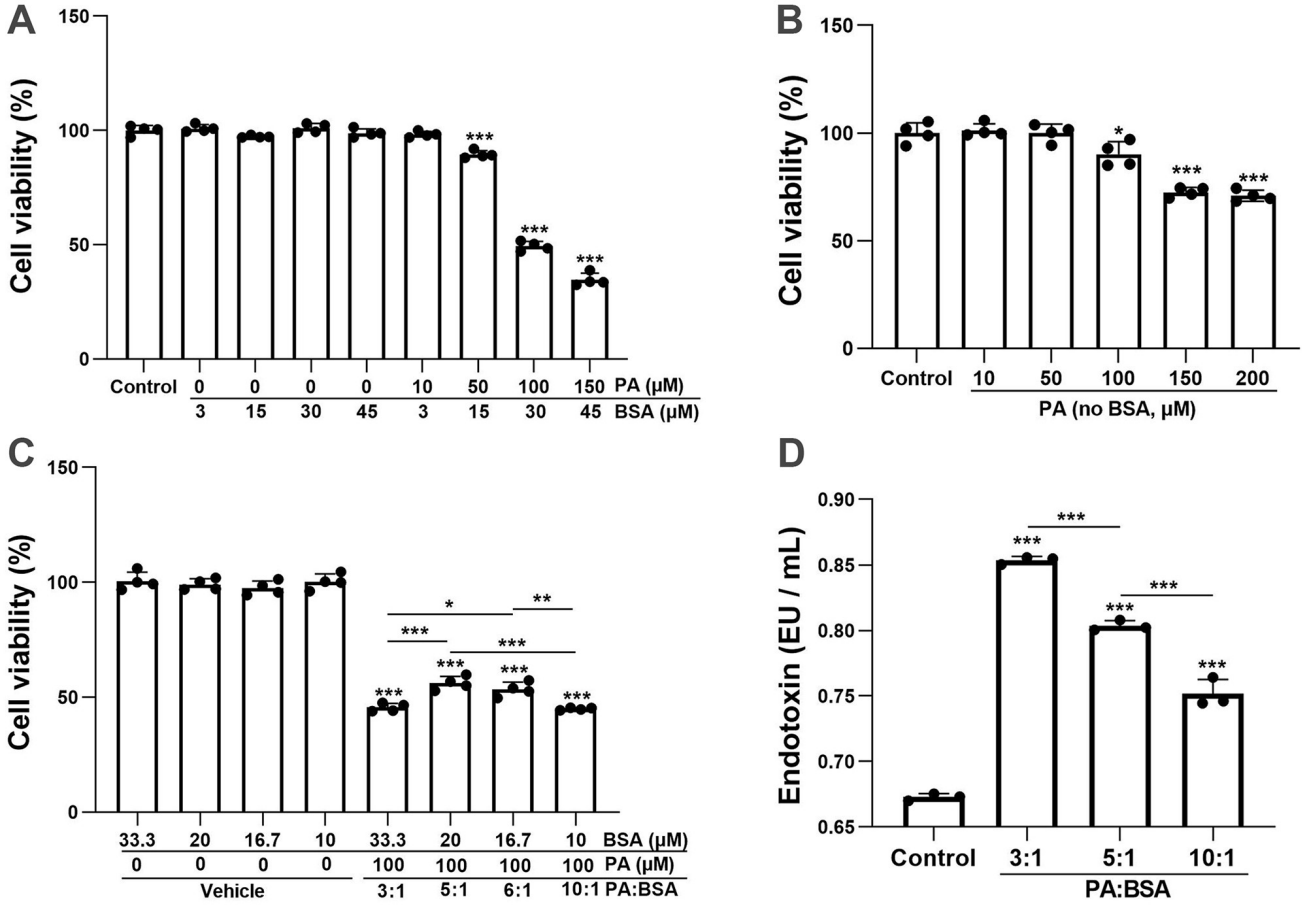
The correlations of PA-BSA binding ratios with BV-2 cell viability. (A) Cell viability of 24 h treatment with different concentrations of PA. The PA to BSA ratio is 3.3:1 for all the concentrations. (B) Effects of different concentrations of PA (no BSA) on cell viability for 24 h. (C) 100 μM PA was co-administered with 33.3 μM (3:1), 20 μM (5:1), 16.7 μM (6:1), and 10 μM (10:1) BSA to BV-2 cells for the evaluation of cell viability. (D) Endotoxin levels of the control group (DMEM without FBS), the PA: BSA groups (3:1, 5:1, and 10:1) in the medium. *p < 0.05, **p < 0.01, ***p < 0.001 by one-way ANOVA with Dunnett’s multiple comparisons test compared with the control group or followed Tukey’s multiple comparisons test between the groups as indicated.

To determine the toxic effect of PA on BV-2 cells, the cell viability after 24 h of treatment with PA-BSA (3:1) was analyzed using the CCK-8 method. As shown in Fig 3A, when the concentration of BSA was under 45 μM, no significant effect on BV-2 cell viability was seen. The same was seen when cells were treated with 10 μM PA. Compared to the BSA-only groups with the corresponding concentration of BSA, 50 μM, 100 μM, and 150 μM PA-BSA treatment reduced cell viability by 8%, 51%, and 67% (*p* < 0.001), respectively (Fig 3A). To determine if the cell viability effects were from PA alone, cells were treated with various concentrations of PA (no BSA) for 24 h. A slight but significant decrease in cell viability by 9.8% (*p* < 0.05) was seen at 100 μM. Cell viability decreased by 28% (*p* < 0.001) and 30% (*p* < 0.001) at 150 μM and 200 μM (Fig 3B). As BSA has the ability to absorb intracellular lipids [19], we hypothesized that redundant BSA (leftover after PA conjugation) may be a significant contributor to microglial death. To evaluate this effect, we reduced the BSA content combined with 100 μM PA to 33.3 μM, 20 μM, 16.7 μM, and 10 μM (a PA to BSA ratio of 3:1, 5:1, 6:1, and 10:1, respectively). BV-2 cells were treated for 24 h with four proportions of PA-BSA and four corresponding vehicle groups. As with the previous result, 100 μM PA with all four proportions of BSA resulted in significant cell death in comparison to the corresponding vehicle groups (Fig 3C). Putting this together, although BSA alone did not affect BV-2 cell viability, when reducing the BSA content in PA-BSA solutions from 3:1 to 5:1, a marked increase in cell viability by 11% (*p* < 0.001) was seen. To our surprise, reducing BSA content in PA-BSA solutions from 5:1 to 10:1 did not further increase cell viability. On the contrary, it greatly decreased cell viability, by 11% (*p* < 0.001). The effect may be attributed to the solubility of PA, as when 10:1 was used, PA was found to form precipitates directly after coupling with BSA. These precipitates can cover the surface of the BV-2 cells, resulting in a higher rate of cell death. To understand the environment around the cells, we detected the levels of endotoxin in the culture medium. The amount of endotoxin was 0.75 EU/mL, 0.80 EU/mL, and 0.85 EU/mL in the PA-BSA groups (10:1, 5:1, and 3:1), whereas there was only 0.67 EU/mL endotoxin in the serum-free medium (Fig 3D).

### A PA to BSA ratio of 5:1 may be the best to use in microglial inflammation studies

**Fig 4:**
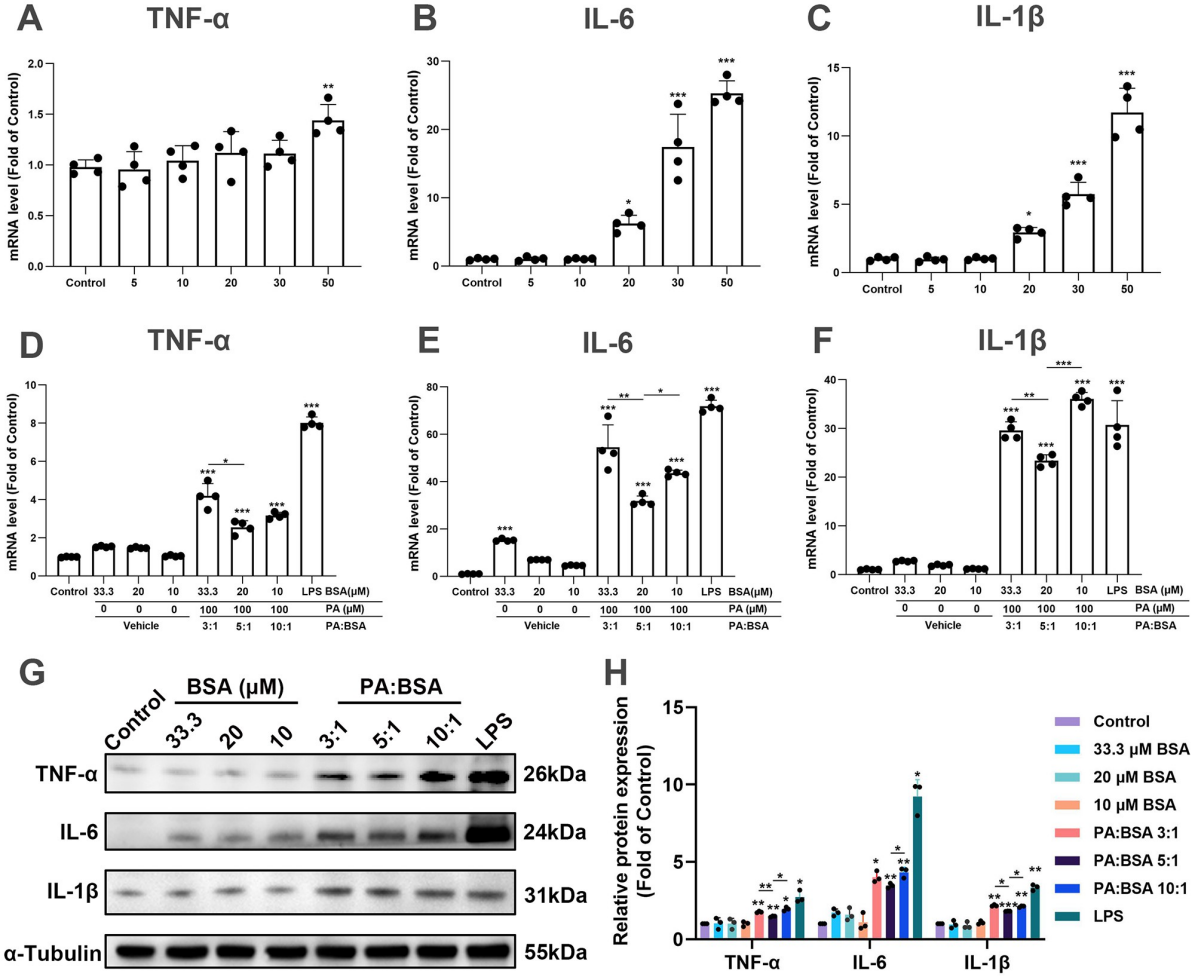
Effect of three PA to BSA binding ratios on the expression levels of inflammatory cytokines in BV-2 cells. (A-C) TNF-α, IL-6, and IL-1β levels were analyzed after incubating cells with 5 μM, 10 μM, 20 μM, 30 μM, and 50 μM PA-BSA for 24 h. (D-E) TNF-α, IL-6, and IL-1β levels were analyzed after incubating cells with a PA-BSA solution of 100 μM PA with 33 μM (3:1), 20 μM (5:1), 10 μM (10:1) BSA, and LPS (1 μg/mL) for 24 h. (G, H) Representative Western blots for the protein expression levels of TNF-α, IL-6, and IL-1β. *p < 0.05, **p < 0.01, ***p < 0.001 by one-way ANOVA with Dunnett’s multiple comparisons test compared with the control group or followed Tukey’s multiple comparisons test compared with the vehicle groups or between the groups as indicated.

To determine the threshold concentrations of PA-BSA causing the elevation of inflammatory cytokines. Cells were treated with PA-BSA (5:1) at concentrations of 5, 10, 20, 30, and 50 μM for 24 h. We found that the thresholds for PA to induce the mRNA expression of TNF-α, IL-6, and IL-1β were 30 μM, 10 μM, and 10 μM, respectively (Fig 4A–4C). To determine whether PA was responsible for the proinflammatory reaction, TNF-α, IL-6, and IL-1β levels were analyzed after incubating cells with a PA-BSA solution of 100 μM PA with 33 μM, 20 μM, or 10 μM BSA for 24 h. As expected, the inflammatory response decreased in a dose-dependent manner with the decrease in BSA concentration. Compared to the vehicle groups, the PA-BSA 3:1, 5:1, and 10:1 groups all showed elevated levels of TNF-α, IL-6, and IL-1β (Fig 4D–4F). Among them, the 5:1 group exhibited the lowest inflammatory profile. When compared to the 3:1 and 10:1 groups respectively, the expression of TNF-α in the 5:1 group decreased by 39% (*p* < 0.05) and 20% (*p* = 0.71), of IL-6 by 42% (*p* < 0.01) and 27% (*p* < 0.05), and of IL-1β by 21% (*p* < 0.01) and 54% (*p* < 0.001). The 10:1 group, containing the lowest amount of BSA, still exhibited a strong inflammatory response and showed the highest level of IL-1β expression.

We further investigated whether the protein expression level followed the expression pattern of mRNA. As shown in Fig 4G and 4H, compared with the corresponding BSA concentration, the protein expression levels of all three inflammatory factors were significantly increased after three proportions of PA-BSA treatment. Similar to the mRNA expressions, the 5:1 group exhibited the lowest inflammatory protein expression. When compared to the 3:1 and 10:1 groups respectively, the protein expression of TNF-α in the 5:1 group decreased by 17% (*p* < 0.01) and 25% (*p* < 0.05), IL-6 by 14% (*p* = 0.09) and 20% (*p* < 0.05), and IL-1β by 17% (*p* < 0.05) and 14% (*p* < 0.05). Based on these results, it can be concluded that the PA to BSA ratio of 5:1 resulted in the lowest expression levels of inflammatory cytokines. This ratio also induced the lowest degree of BV-2 cell death, which may indicate that PA induced BV-2 cells inflammation may result from cell death.

### PA-BSA or BSA alone elevate the cytosolic LPS levels that may lead to pyroptosis

**Fig 5:**
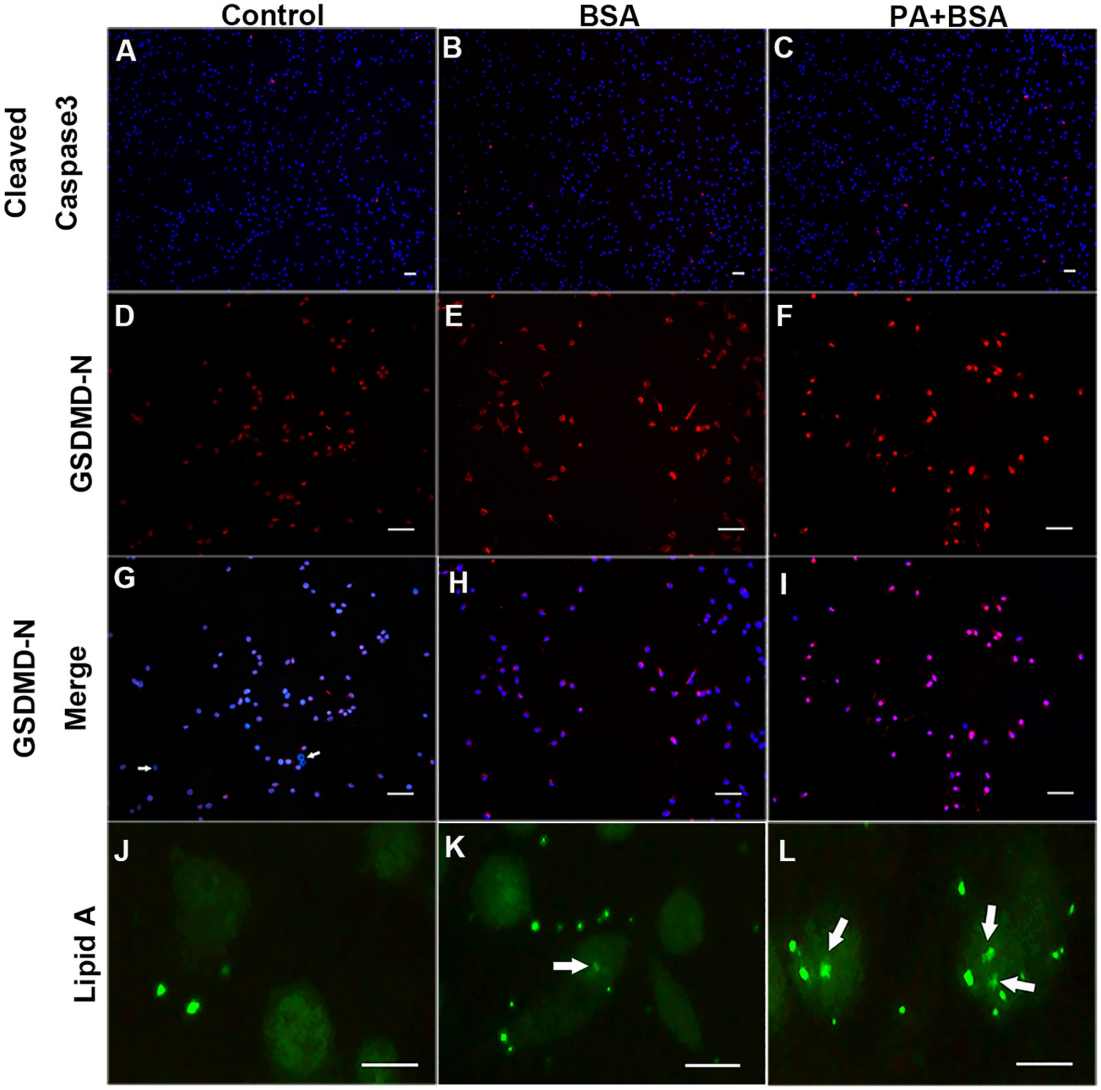
PA-BSA or BSA alone increased cytoplasmic LPS levels and induced pyroptosis. BV-2 cells were treated with BSA and PA for 4 h. (A-C) Cleaved-caspase3 (red) merged with DAPI (blue). (D-F) GSDMD-N immunofluorescence staining. (G-I) GSDMD-N (red) merged with DAPI (blue). Arrows indicate GSDMD-N negative cells. (J-L) Lipid A immunofluorescence staining. Arrows indicate Lipid A positive components inside the cell. Scale bars: A-I 30 μm, J-L 15 μm.

**Fig 6:**
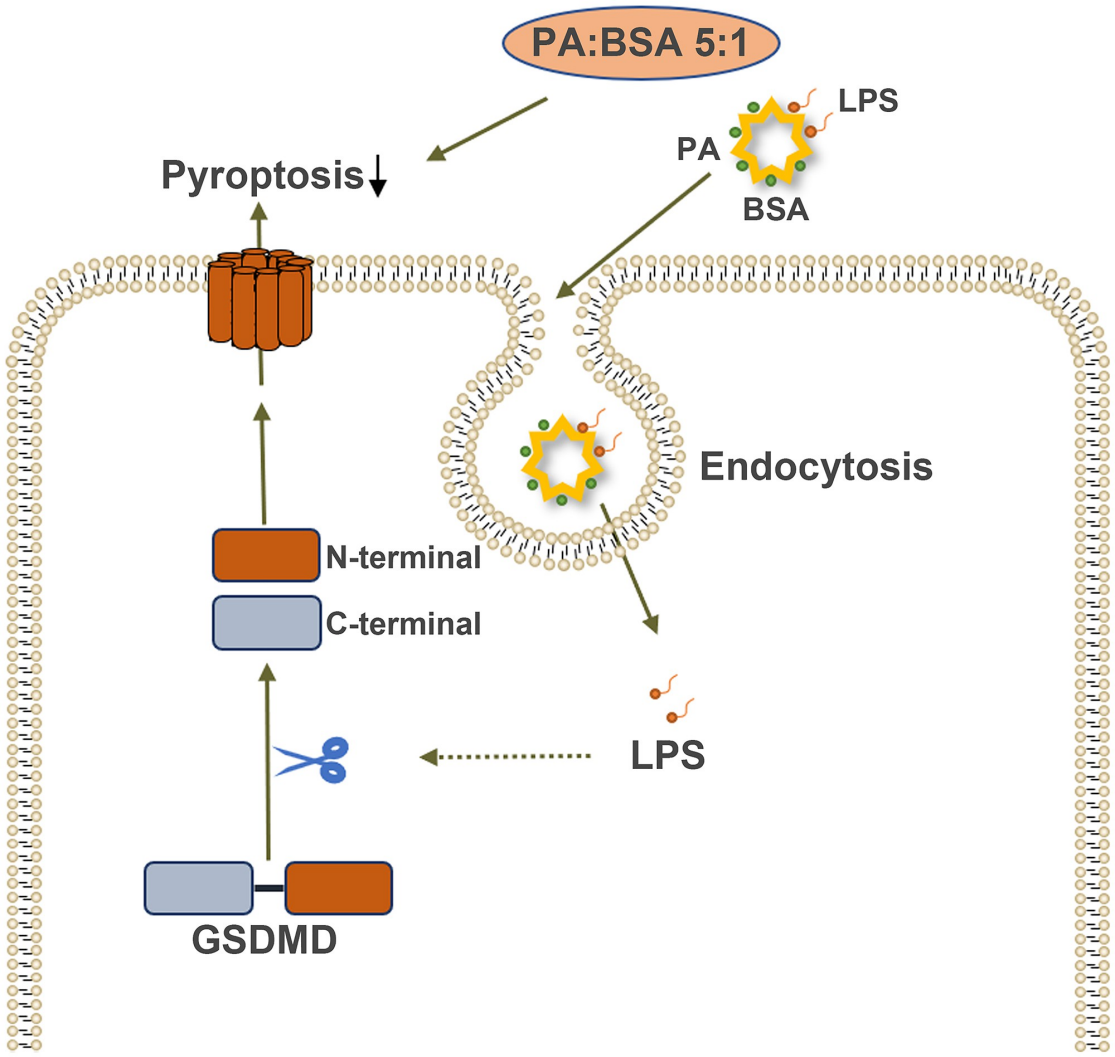
The model of PA/BSA-induced cell death was indicated.

To analyze the reason why PA gave rise to massive cell death in such a short time, we relied on immunohistological examinations after treating cells with PA-BSA for 4h for detecting cleaved-caspase-3, the N terminal of GSDMD (GSDMD-N), and lipid A of LPS (Fig 5). Apoptosis seemed to be a minor event, as we found only 0.83% ± 0.47%, 1.41% ± 0.22%, and 1.84% ± 0.75% apoptotic cells in the control, the BSA group, and the PA-BSA group, respectively (Fig 5A–5C). Many red cells undergoing apoptosis did not have nuclei, indicating that these cells were activated much earlier. There were GSDMD-N expressions in all the three groups (Fig 5D–5F). A few GSDMD-N negative cells were found in the control, indicating that these cells were not undergoing pyroptosis (Fig 5G). Surprisingly, BSA alone was able to induce a strong GSDMD-N signal (Fig 5E). When BSA was coupled with PA, this pyroptotic signal became stronger (Fig 5F). Since TLR4 expressions were nearly the same among the three BSA brands, the environmental LPS might not play a major part in this pyroptotic process. We stained lipid A for detecting intracellular LPS. In the control, cytosolic LPS appeared to be a rare case (Fig 5J); however, small and blur spots were found inside the cells in the other groups (Fig 5K and 5J). PA+BSA produced more LPS in the cytosol, which caused severe non-canonical pyroptosis (Fig 5L). The model of PA/BSA-induced injury was summarized in Fig 6.

## Discussion

In the current study, we evaluated the preparation of BSA-PA complex and further verified the effects in a microglial model. Taking into consideration the solubility of PA, the proinflammatory nature of BSA, and interference due to the solvent, we found the sodium palmitate to be the best form for the administration of palmitic acid and 5:1 (PA:BSA) to be the best binding ratio for studying inflammation in BV-2 microglia.

The brand of BSA used may be of great importance in when complexing FFA with BSA. In our pilot study, we repeated the work of Tracy et al., still obtaining results that indicated a strong immune response due to PA treatment. We believe the discrepancy may be due to difference in BSA brand and type used (not mentioned by Tracy et al.) and the quenching effect of the ethanol. It is also reported that cell death was significant when cells were treated with 250 μM palmitate in the presence of 75 μM BSA (3.33:1 palmitate to BSA) but became undetectable as BSA was increased to 150 μM (1.67:1) and 225 μM (1.11:1) [29]. In our experiments examining cell viability after increasing the BSA concentration to 150 μM, we found cell death still detectable. These differences may also be related to the brand or type of BSA. It should be noted that there are currently approximately 50 types of BSA available from just one company (Merck) [19].

In our experiments, we used low endotoxin BSA because it is the most commercially available form of BSA, and because even the so-called “Endotoxin-free” BSA was proved to still have proinflammatory effects on cells [30]. Another reason is that the preparation of FFA-free BSA requires charcoal filtration, which inevitably results in the presence of residual endotoxin. We chose to use sodium palmitate instead of palmitic acid in order to increase solubility and to minimize the side effects caused by the solvent (ethanol or isopropanol). Our results have shown that ethanol is harmful to microglia and dampens TNF-α, IL-6, and IL-1β expression, which is consistent with the phenomenon observed in the literature by other groups [22]. The incubation temperature for PA with BSA usually falls within the range of 37°C to 70°C; however, BSA has been reported to be partially degraded after 2 min at 50°C and appeared to form aggregates [31]. Accordingly, we chose to use 37°C in this study.

At the beginning of the study, we postulated that pH may be an important variable when incubating PA with BSA, as we observed a change in pH during the reaction. On review, it appears that pH effects are negligible for cell viability because the levels may be buffered by the incubator.

BSA is a proinflammatory substance, and from our results the ratio of 10:1 PA to BSA can be used to minimize the inflammatory influence of BSA; however, this also results in a rather low solubility. The avoidance of PA to BSA ratios greater than 5:1 has been suggested in order to reduce artifacts caused by low solubility [6].

To our knowledge, this study shows for the first time that albumin increases cytosolic LPS and promotes microglial pyroptosis. PA-BSA has recently been shown to facilitate LPS entering the cell [32]. Furthermore, the lipotoxicity of PA can also come from BSA-related LPS transfer. This may explain the contradictory observation that PA can increase neuroprotective orexin-1 receptors and be proinflammatory at the same time [33]. Ranaivo and Wainwright demonstrate that albumin activates microglia and significantly increases IL-1β levels [34]. We now know that IL-1β is released from cells by going through the GSDMD-formed pores, and this mechanism was predicted by a previous study in 2006 [35]. Thus, their albumin effects could result from pyroptotic cell lysis.

Although we found an optimal ratio for coupling BSA and PA, we cannot rule out the possibility of unbound BSA serving as a lipid tank, which can trap a significant amount of fatty acids, preventing them from exerting their normal physiological functions. It is also possible that LPS can compete binding the sites on albumin with fatty acids, as one of the major toxic components of LPS is lipid A. Oleic acid has been well-characterized to alleviate PA-induced cytotoxicity. We think it might be because unsaturated fatty acids have better affinity than PA, less LPS, therefore, would gain access to the cell. We were able to show in this experiment that PA indirectly induces microglial inflammation through affecting cell viability, and in future experiments we will aim to further investigate the relationship between PA-induced microglial death and inflammation.

## Supporting information

S1 Raw images (PDF)

S1 Dataset Raw data of this article. (ZIP)
