# Supplementary figures and images for: Palmitate lipotoxicity is closely associated with the fatty acid-albumin complexes in BV-2 microglia
Source: PLoS One. 2023 Apr 20;18(4):e0281189. doi: 10.1371/journal.pone.0281189 (PMC10118109; doi:10.1371/journal.pone.0281189)

**Fig 2**

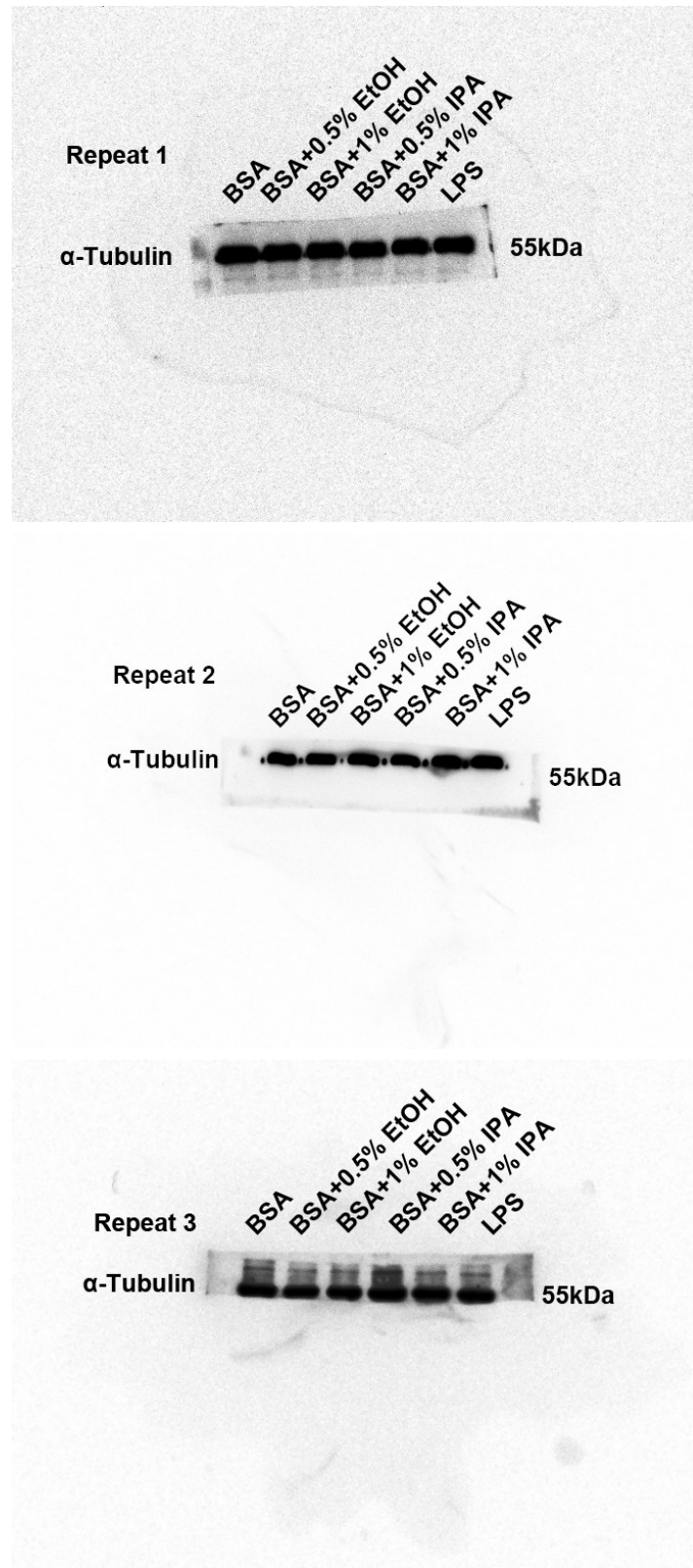

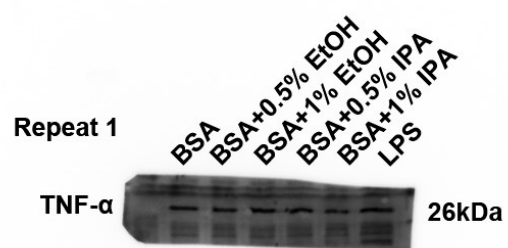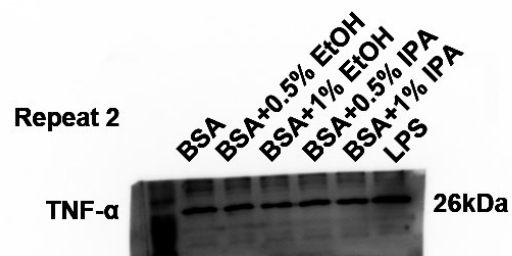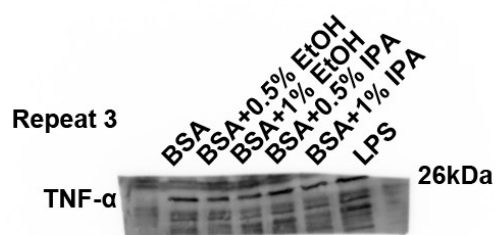

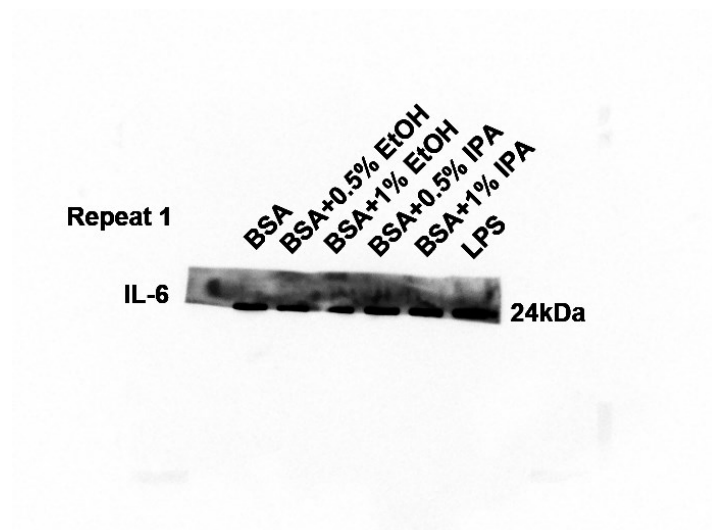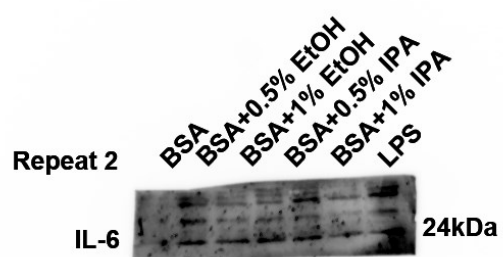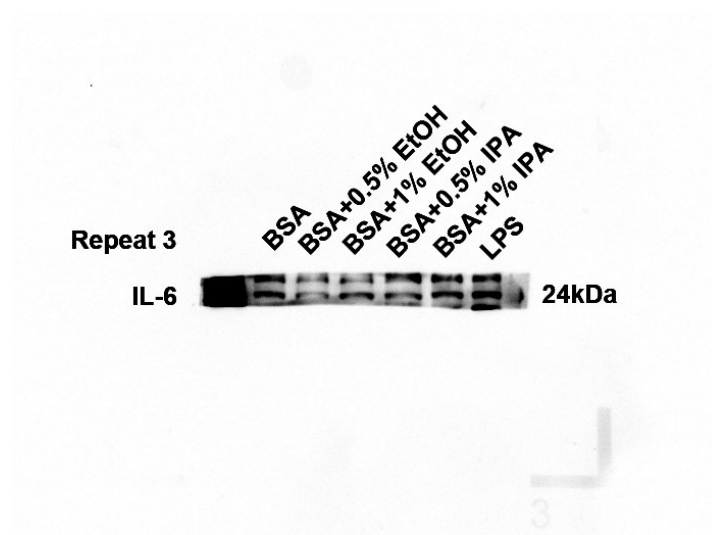

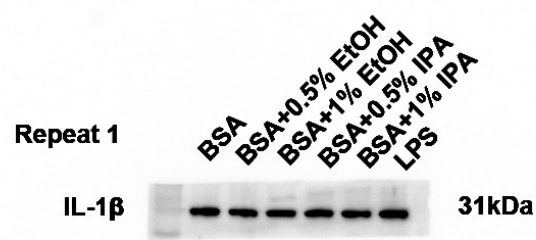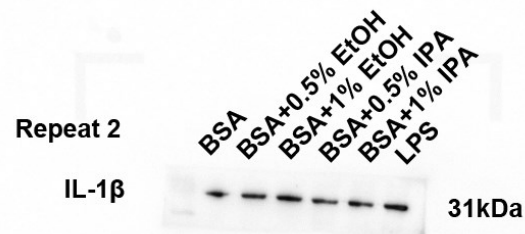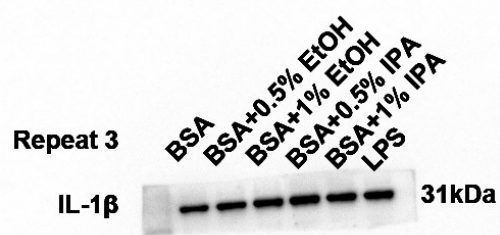

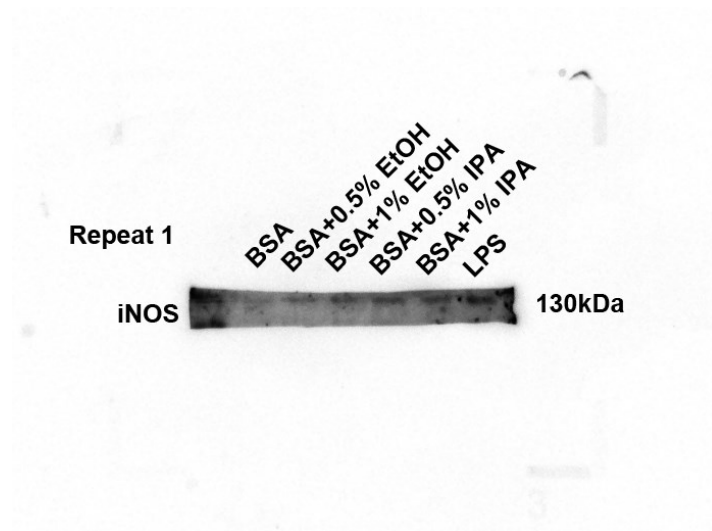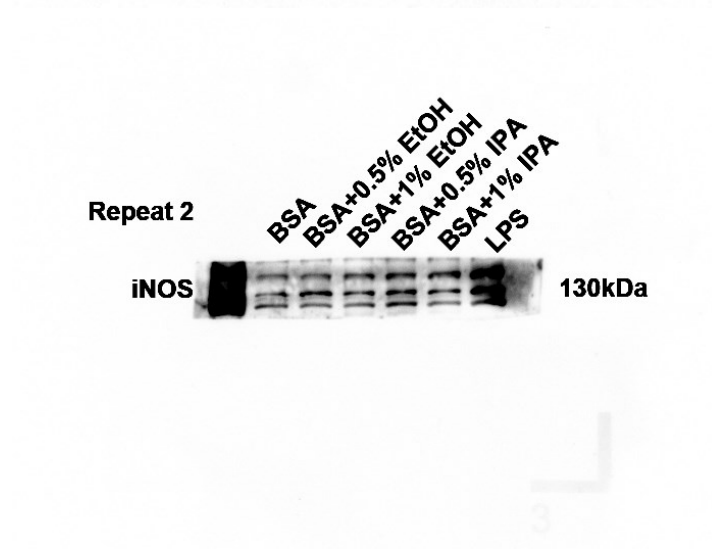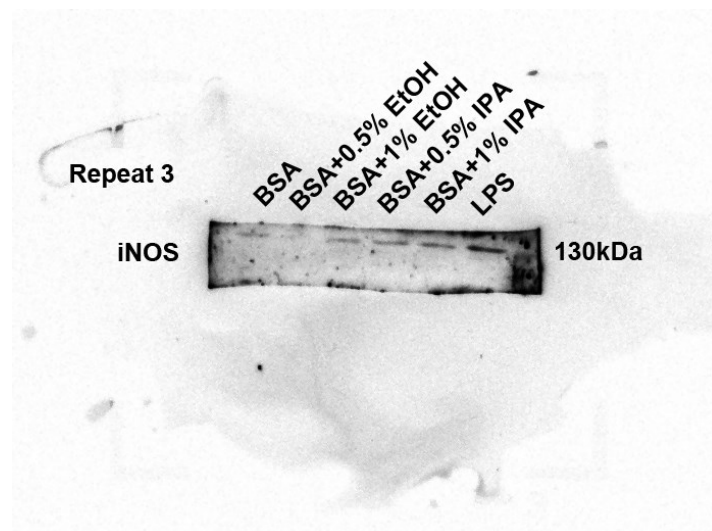

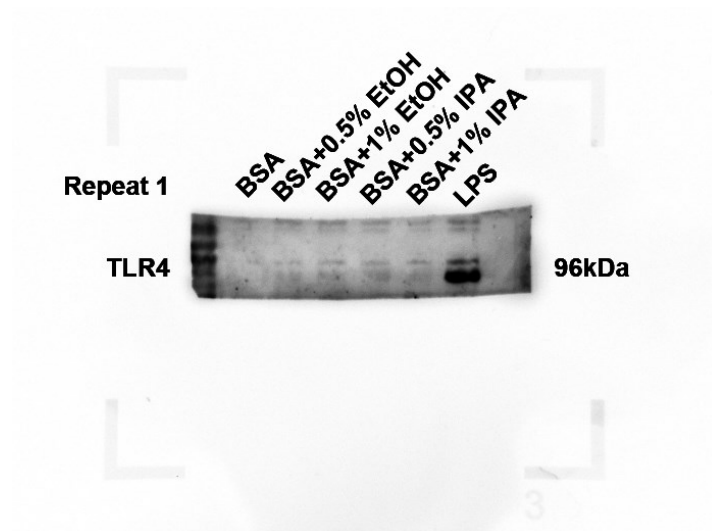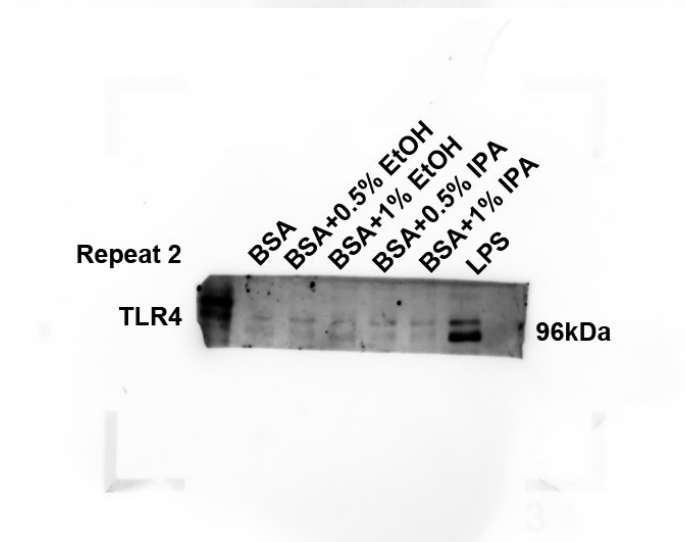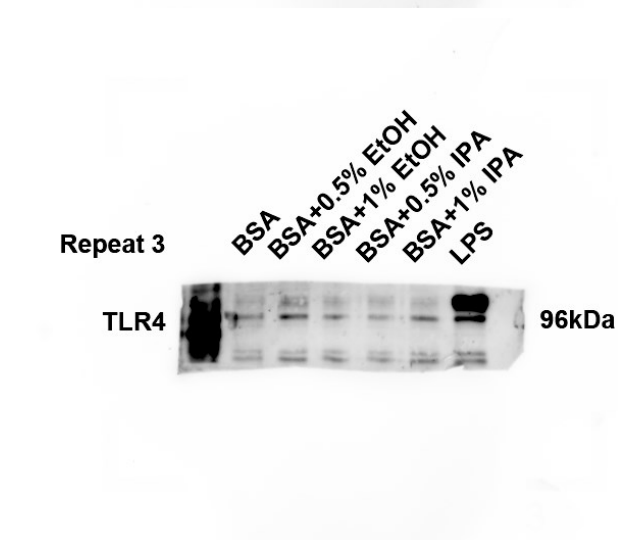

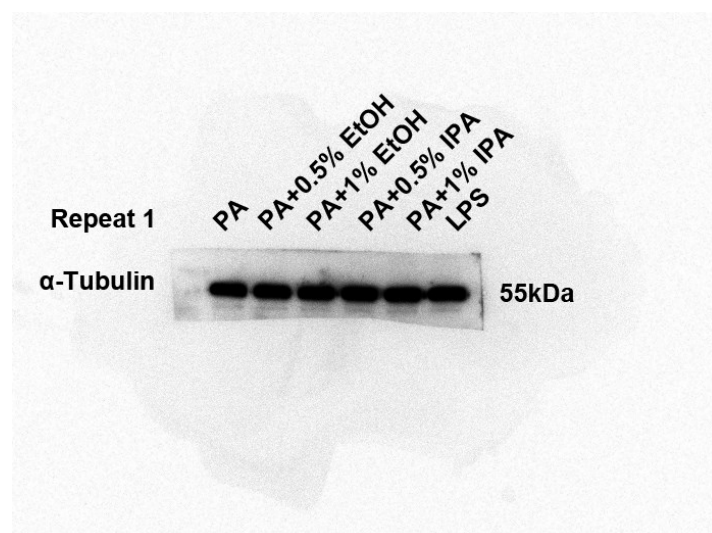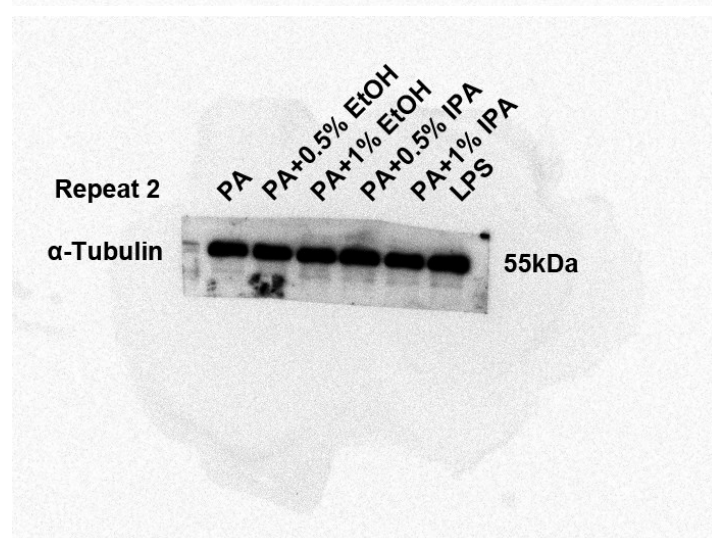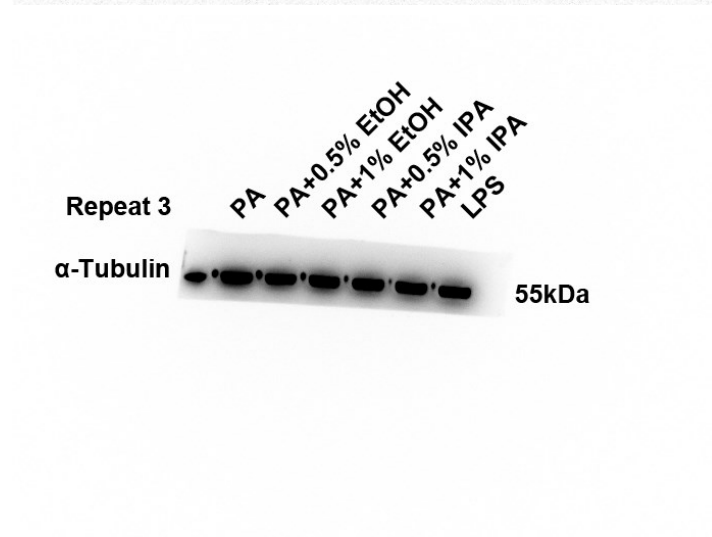

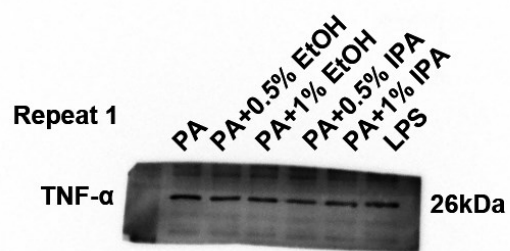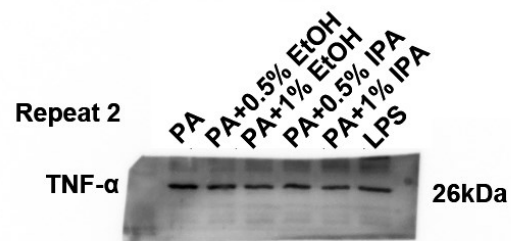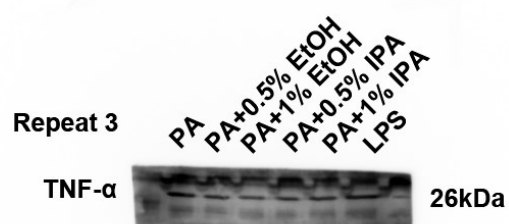

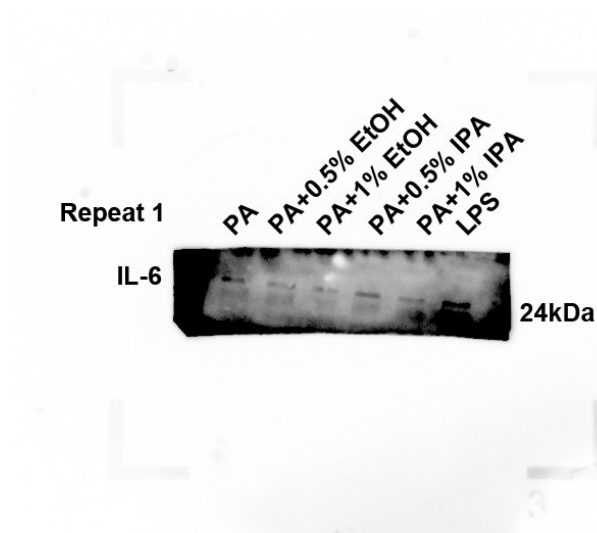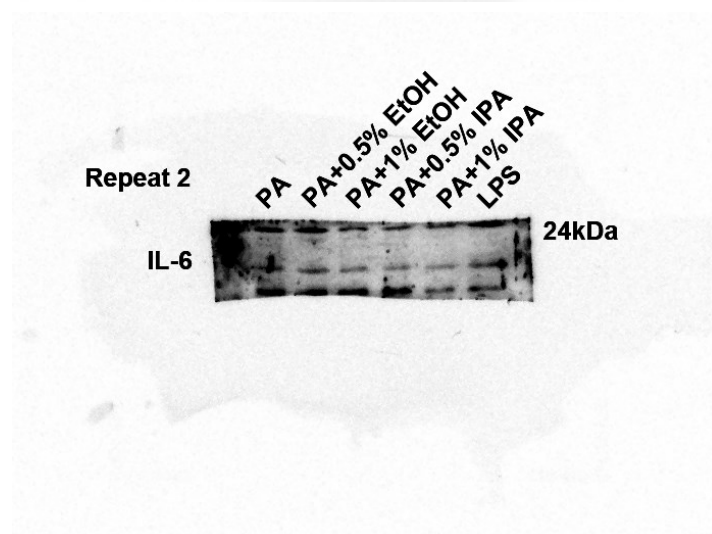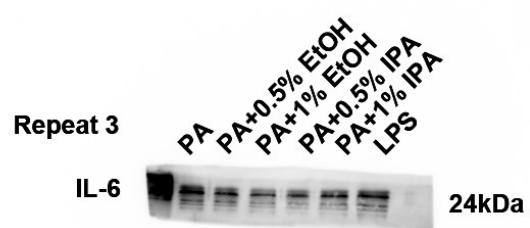

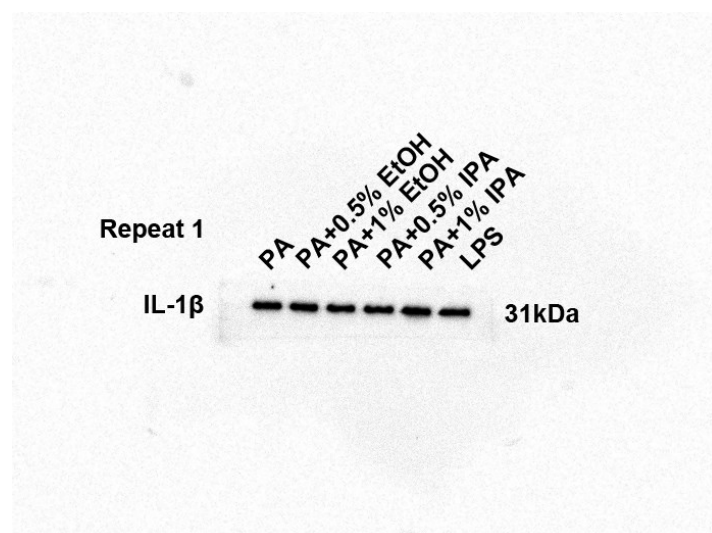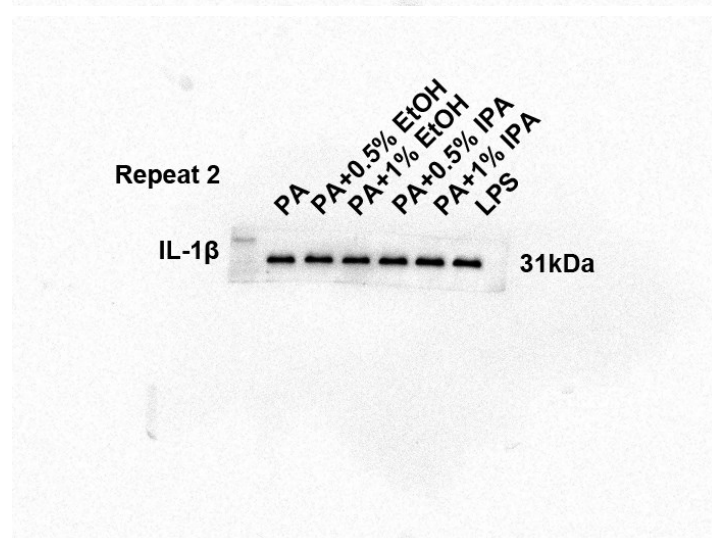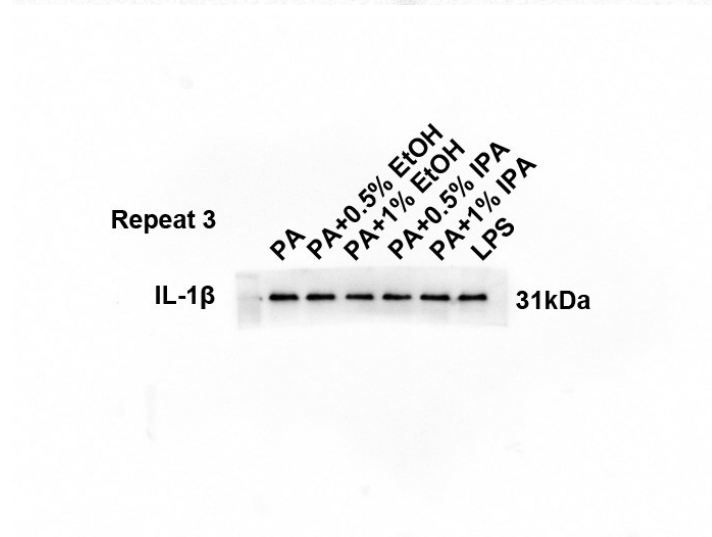

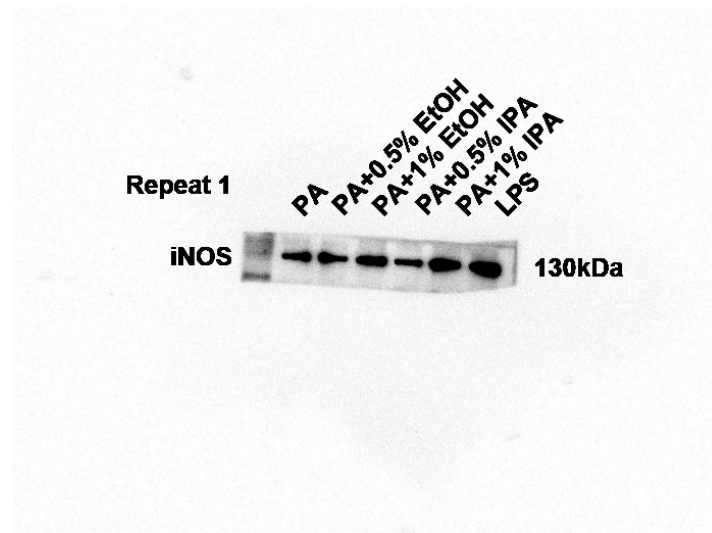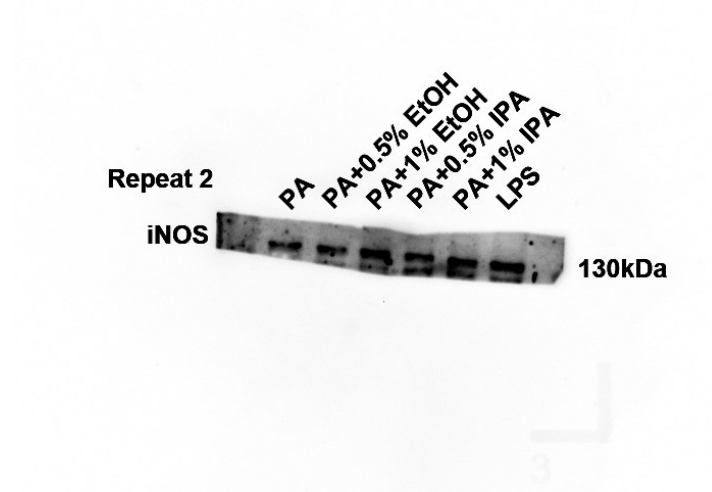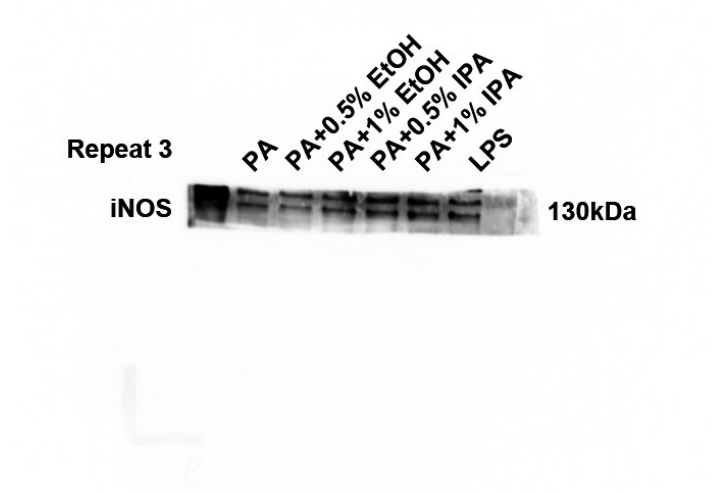

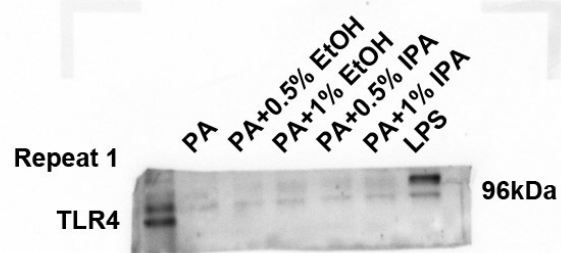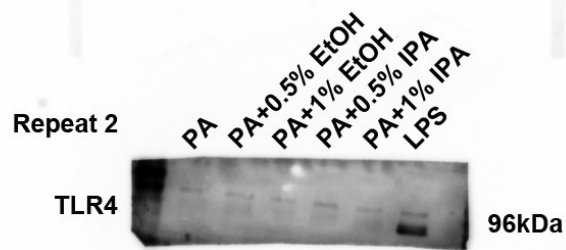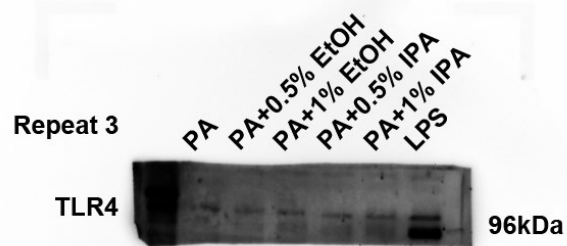

Fig 4

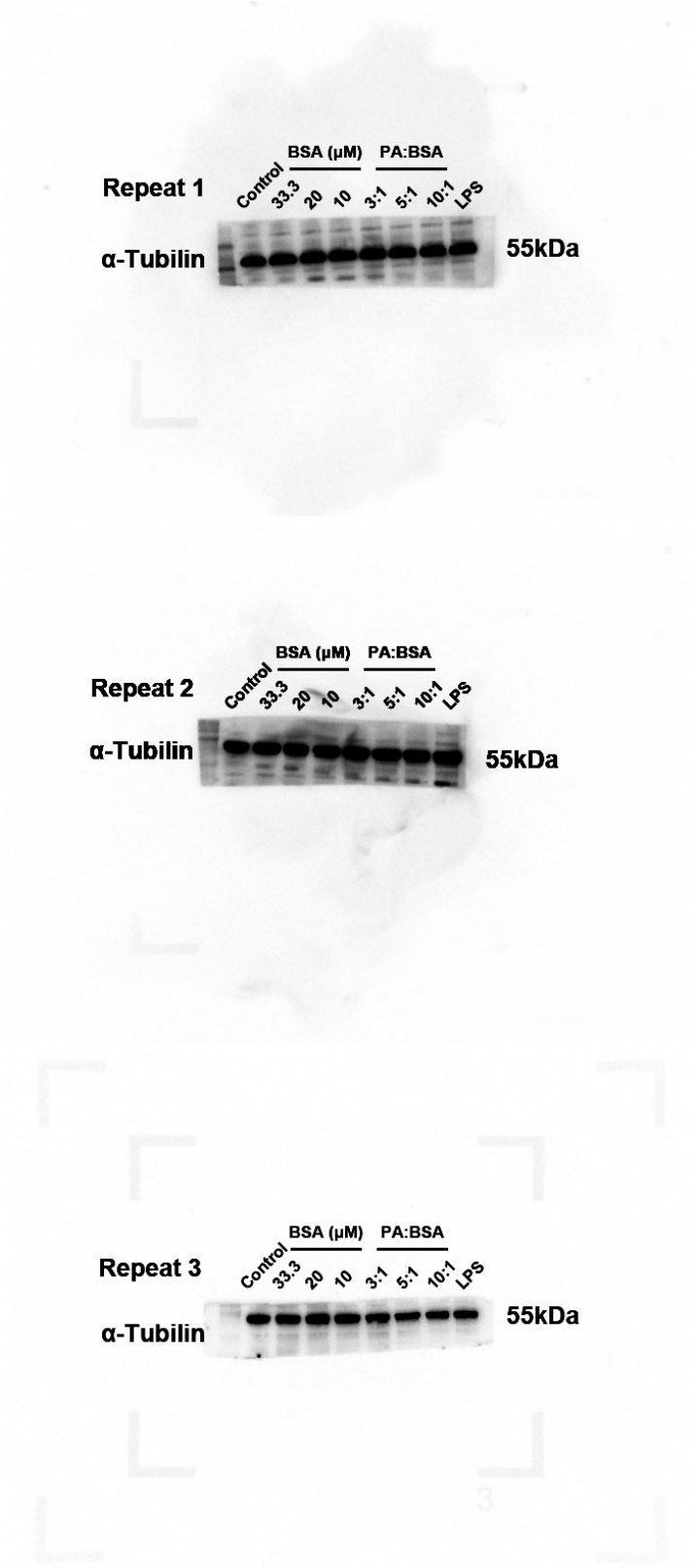

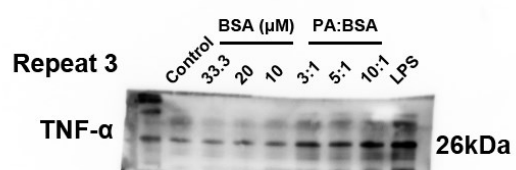

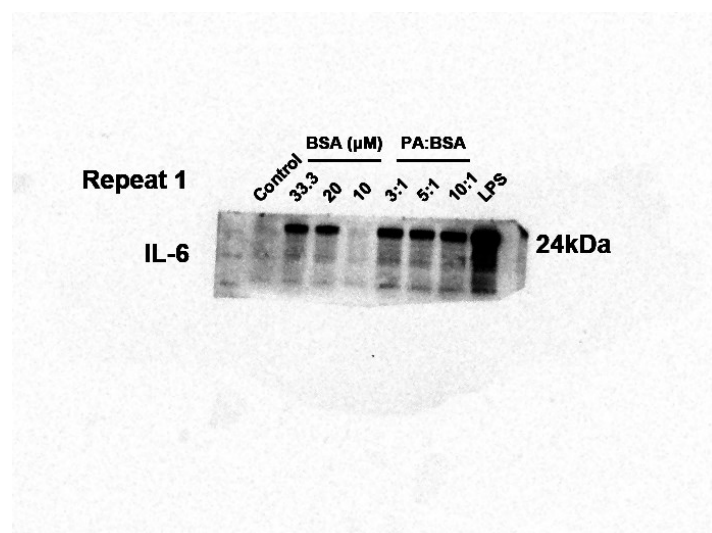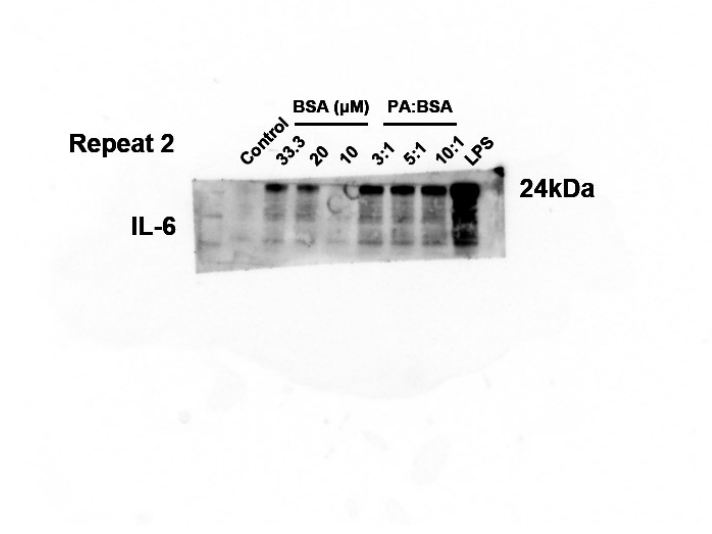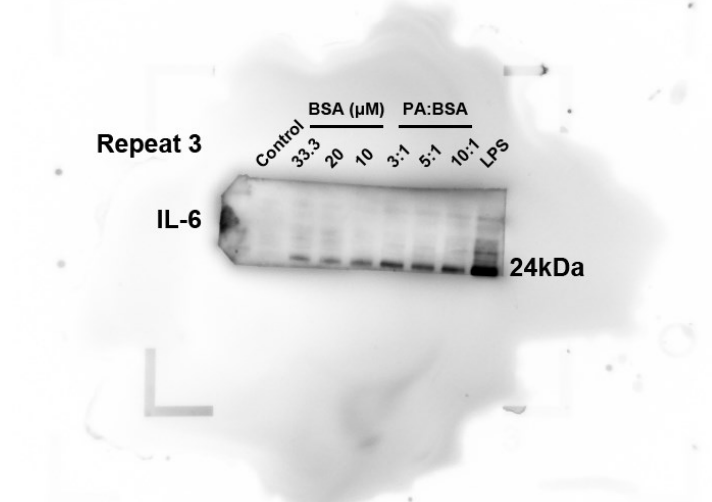

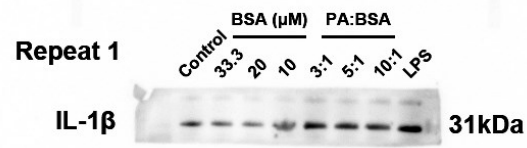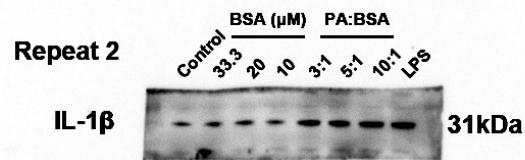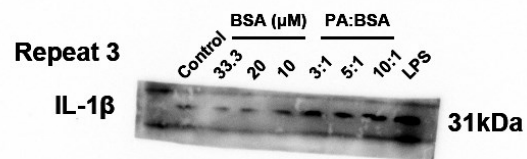

Supplement: S1 Raw images — (PDF) [file pone.0281189.s001.pdf]
